# Challenges of Renal Function Assessment in Breast Cancer Patients Treated With Abemaciclib: A Case Report

**DOI:** 10.7759/cureus.67714

**Published:** 2024-08-25

**Authors:** André Ferreira, José Brito da Silva, Maria Teresa Chuva, José Maximino Costa, Deolinda Pereira

**Affiliations:** 1 Department of Nephrology, Unidade Local de Saúde Viseu Dão-Lafões, Viseu, PRT; 2 Department of Nephrology, Instituto Português de Oncologia do Porto Francisco Gentil EPE, Porto, PRT; 3 Department of Medical Oncology, Instituto Português de Oncologia do Porto Francisco Gentil EPE, Porto, PRT

**Keywords:** case reports, breast neoplasms, radioisotope renography, cystatin-c, creatinine, glomerular filtration rate, kidney function tests, abemaciclib

## Abstract

Abemaciclib, a cyclin-dependent kinase 4/6 (CDK4/6) inhibitor used for hormone-receptor-positive and human epidermal growth factor receptor 2 (HER-2)-negative breast cancer, can lead to elevated serum creatinine without implications on the true renal function. Although clinical trials have shown no increase in other kidney function biomarkers, this may still represent a challenge in cancer patients. We report a case of a 74-year-old female who presented with creatinine and cystatin-C elevation during treatment with abemaciclib without an equivalent decrease in measured glomerular filtration rate (GFR) with renal scintigraphy. The confirmation of adequate kidney function allowed for the maintenance of treatments that would otherwise be limited by renal impairment. Healthcare providers should be aware of abemaciclib's effect on serum creatinine but should not eliminate the possibility of actual kidney injury. Alternative biomarkers for GFR assessment are recommended, although the usefulness of cystatin-C in patients receiving abemaciclib should be investigated in greater depth.

## Introduction

Breast cancer is the most common cancer affecting European women, with an incidence of 144.9/100,000 in 2018 and mortality of 15.2/100,000 in 2012; however, both incidence and mortality rates are declining [1,2]. Recent therapeutical advances have warranted updates in clinical practice guidelines. In patients with hormone-receptor-positive and human epidermal growth factor receptor 2 (HER-2)-negative metastatic breast cancer without imminent organ failure, current standard first-line therapy includes a combination of endocrine therapy, such as aromatase inhibitors or tamoxifen and cyclin-dependent kinase (CDK) 4/6 inhibitors [3]. Abemaciclib, one of these inhibitors, is structurally distinct from palbociclib and ribociclib, showing a greater affinity for CDK4 than CDK6 inhibition, and has different dose-limiting toxicities, mainly fatigue rather than hematological toxicity [4,5]. This CDK4/6 inhibitor also has a benefit in the adjuvant setting, preventing early disease recurrences and reducing the risk of distant metastases in high-risk patients [6]. These indications have led to an increasing use of this drug in clinical practice.

Clinical trials that led to abemaciclib approval showed an increase in serum creatinine (sCr) in up to 95% of patients [7]. This increase can reach nearly 15-40%; however, that percentage can be greater in patients with kidney disease [5,8]. Nevertheless, these changes do not necessarily mean a true decrease in renal function [9], and less than 5% of patients experienced grade 3 or 4 renal toxicity [10]. Abemaciclib is a competitive inhibitor of efflux transporters (mediators of active secretion) of creatinine, such as organic cation transporter 2 and multidrug and toxin extrusion protein [9,11]. This can lead to an increase in sCr, which conversely reflects a decrease in glomerular filtration rate estimated (eGFR) with equations based on sCr. Nonetheless, these changes are not reflected in the measured glomerular filtration rate (GFR) (as determined by iohexol clearance), in the eGFR with equations based on serum cystatin-C (sCysC) nor in the measurement of other kidney function biomarkers - urea, kidney injury molecule 1 or neutrophil gelatinase-associated lipocalin [9,11].

Given the limitations of the eGFR with sCr, biomarkers independent of the active tubular secretion process, such as sCysC, seem to provide a more accurate estimate of renal function in patients on abemaciclib [5].

## Case presentation

We report a case of a 74-year-old female with metastatic breast cancer and a personal history of atrial fibrillation and arterial hypertension for more than 25 years, currently treated with valsartan 160 mg/day and hydrochlorothiazide 12.5 mg/day.

She was diagnosed 10 years before with localized lobular carcinoma of the left breast and was subjected to multimodality treatment (neoadjuvant chemotherapy (four cycles of doxorubicin/cyclophosphamide and four cycles of docetaxel), surgery, adjuvant external radiotherapy, and adjuvant endocrine therapy (anastrozole)). Four years later, the disease relapsed in the form of lumbar bone metastasis, and the patient was treated with local radiotherapy and started on first-line palliative endocrine therapy with exemestane and zoledronic acid. During the following four years, the patient had several disease progression-related events under different therapies (fulvestrant for five months, capecitabine for 11 months, and tamoxifen for nine months). Ten years after diagnosis, the patient began fifth-line palliative treatment with the CDK4/6 inhibitor abemaciclib. Zoledronic acid was never stopped.

At the time of CDK4/6 inhibitor initiation, sCr was 1.25 mg/dL (normal range value 0.6-1.3mg/dL), corresponding to a 45 mL/min/1.73 m^2^ eGFR calculated by the 2021 CKD-EPI creatinine equation, without evidence of proteinuria (stage G3bA1 chronic kidney disease (CKD)). Four weeks after abemaciclib initiation, the sCr level rose to 2.12 mg/dL and got as high as 2.25 mg/dL three months later. Creatinine clearance worsened to 22 mL/min (calculated by the Cockcroft-Gault equation), which would potentially limit therapy with either abemaciclib and zoledronic acid. There was no evidence of hydroelectrolytic, acid-base, or calcium and phosphorus metabolism disorders. Furthermore, serum urea levels never rose above 33 mg/dL during this period. Therefore, despite the rise in sCr, treatment was continued, and the patient was referred for nephrology evaluation.

**Figure 1:**
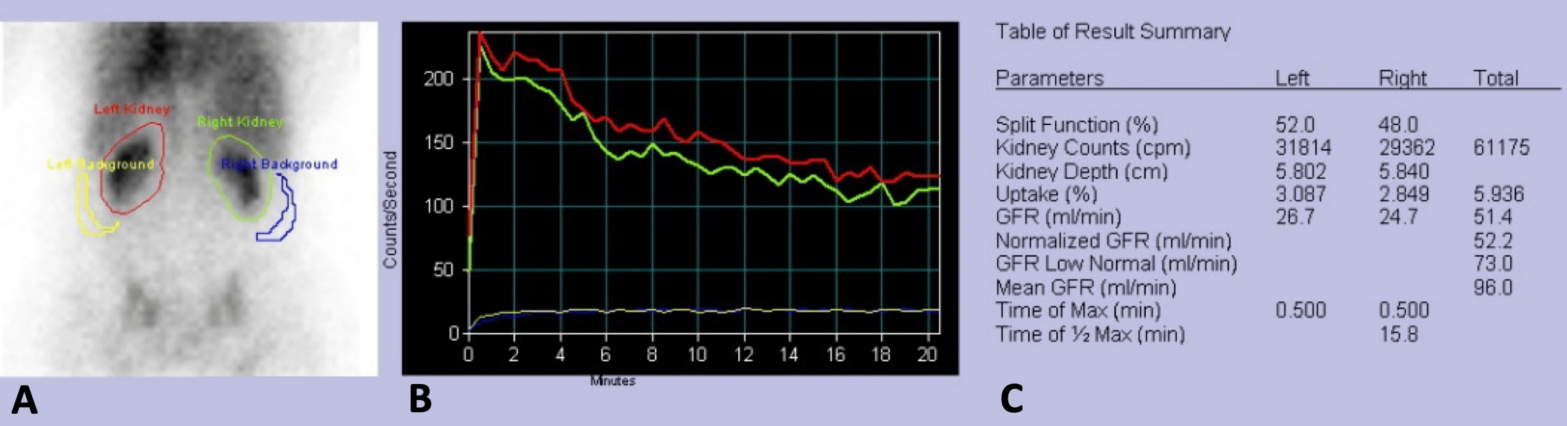
Renal scintigraphic study after intravenous administration of 99mTc-DTPA (technetium-99m-diethylene-triamine-pentaacetate) A static renal scintigraphy image (A), renogram (B), and result summary (C) GFR, glomerular filtration rate

From that evaluation, it was highlighted that there was no history of diabetes mellitus, blood pressure was well controlled (home measurements < 140/90 mmHg), and no new medications had been started. No other causes of acute kidney injury (AKI) were identified. Urinalysis had no clinically relevant changes. Four months after starting the drug, both sCr and sCysC levels were high, corresponding to an eGFR of 28 mL/min/1.73 m^2^ (2021 CKD-EPI creatinine-cystatin equation). In the same month, a radioisotope renography showed a measured GFR of 51.4 mL/min (Figure 1). Given this discrepancy, it was assumed that the patient had only a mild impairment of renal function and should therefore continue treatment.

**Figure 2:**
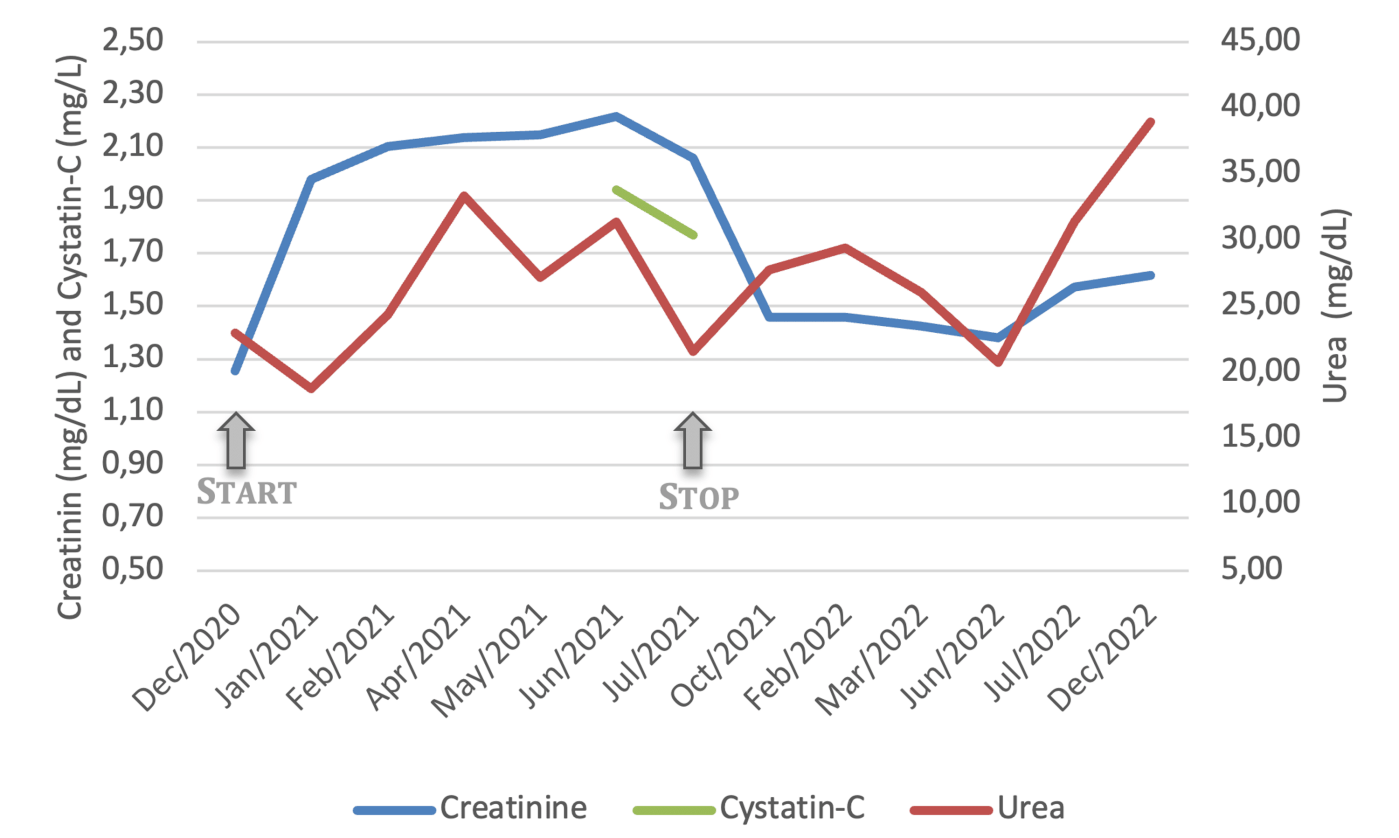
Renal function parameters (serum creatinine, urea, and cystatin-C) evolution from the beginning of treatment with abemaciclib until the last clinical evaluation Arrows indicate the beginning and stopping dates of abemaciclib treatment.

During treatment with abemaciclib, both sCysC and sCr calculated eGFR were equally decreased, disparate from the GFR measured by radioisotope renography (Figure 2). Unfortunately, according to operative follow-up protocols, repeated measurements of sCysC were not performed.

In July 2021, there was evidence of neoplastic disease progression, and abemaciclib was stopped. Despite a trend toward lower values, sCr decreased and stabilized at about 1.5 mg/dL at the beginning of 2022, with a corresponding eGFR of about 37 mL/min/1.73 m^2^, without evidence of proteinuria (stage G3bA1 CKD), representing a worsening in renal function compared to the staging done in December 2020, before prescription of abemaciclib.

Regarding anti-cancer treatment, a sixth-line treatment with oral vinorelbine was started. The patient refused further chemotherapy, and, in December 2022, she was referred to the palliative care service for the best supportive treatment and passed away in November 2023.

## Discussion

This case raises several questions regarding kidney function assessment in cancer patients treated with abemaciclib, in particular: (1) the validity of the available biomarkers for kidney function, (2) the risk of AKI and CKD progression secondary to this drug, and (3) how we can differentiate between the effective or spurious decrease in kidney function in these patients.

Overall, sCr usually increases around two weeks after the start of abemaciclib. It then maintains steady values until the discontinuation of the drug, returning to baseline around 30 days after withdrawal [7,11]. This pattern is explained by abemaciclib’s effect on the inhibition of creatinine tubular secretion, which is not followed by other kidney function biomarkers [9,11].

In our case, sCr elevation was accompanied by sCysC elevation, without a corresponding decrease in GFR measured by radioisotope renography; this behavior of sCysC is not reported in the literature [9,11], but it seems not to correspond to a true AKI as effective GFR was measured by radioisotope renography and found to be close to the previously known baseline kidney function of the patient. Noticeably, further measurements of sCysC would have helped to better ascertain the possibility of a true versus spurious AKI, as a reassessment of the GFR by radioisotope renography could have also been considered to strengthen our findings.

Regarding GFR measurement methods, studies have shown that both in the general population and in patients with CKD, estimating GFR with sCr is an inferior method when compared to using both sCr and sCysC [12]. However, there are several caveats to this combined strategy. For a start, validation of the sCysC-based equations in cancer patients is still expected. In fact, data regarding sCysC use in estimating kidney function in cancer patients is conflicting, with studies reporting different accuracies depending on tumor subtype and timing of assessment [13]. It is also important to consider that sCysC can be influenced by factors commonly present in cancer patients, such as obesity, inflammation, or therapy with steroids, among others [14]. As such, it seems plausible that sCysC may not be completely valid as a surrogate biomarker of renal function in patients with cancer, and its use should be interpreted with caution, particularly in the case of therapy with drugs such as abemaciclib. GFR measured by radioisotope renography could be used in dubious cases, as in the one presented.

In another matter regarding the presented case, we have only a single measurement of GFR by radioisotope renography, which difficulties the assessment of GFR evolution during therapy. However, it is noticeable that sCr did not return to baseline values after the interruption of abemaciclib. Being left without any factor interfering with creatinine tubular secretion, several hypotheses can be made considering either the drugs in use or the patient comorbidities. We propose the following possible explanations:

Drug-related nephrotoxicity because of abemaciclib: Although sCr’s rise during abemaciclib treatment seems to be attributable to the inhibition of tubular secretion, nephrotoxicity of this drug is also possible. Case reports suggest that there may be a risk of AKI associated with abemaciclib [15], and, in a series of cases, biopsy-proven pathologic features of AKI were confirmed [16]. This highlights the difficulty in assessing renal function and managing these patients, particularly if they already have CKD or have other kidney injury events/nephrotoxic drugs.

Drug-related nephrotoxicity from other drugs besides abemaciclib: In patients with baseline CKD, treatment with zoledronic acid might cause acute tubular necrosis, being a risk factor for continued deterioration of renal function [17] (especially when considering repeated cycles). Real-world evidence shows that combining abemaciclib with bisphosphonates is safe as the possible effect of abemaciclib on sCr is not expected to favor bisphosphonate toxicity [18]. Furthermore, the patient was under valsartan, an angiotensin receptor blocker, which may directly cause AKI or may lower the threshold for AKI because of other insults, in relation to its mechanism of action.

Progression of CKD associated with arterial hypertension: Despite the patient having long-standing hypertension, her arterial blood pressure was well-controlled in the outpatient setting, rendering it less likely to participate in CKD progression. Notwithstanding, any patient with any degree of CKD is expected to worsen within due time, although there should be a plausible cause for a steeper decrease in GFR, which was not evident in this case.

GFR’s decrease associated with aging: kidneys suffer structural and functional alterations related to aging, such as increased nephrosclerosis. Among healthy individuals, GFR declines steadily at a rate of approximately 6 mL/min/1.73 m^2^ per decade [19], which might be accelerated by other comorbidities, such as long-standing high blood pressure.

As measured GFR showed no true AKI while on treatment with the denoted drugs, we find it unlikely for the same drugs to be the major cause of the kidney function deterioration after abemaciclib cessation. As such, it seems more plausible that CKD progression was attributable to the patient’s aging and comorbidities (independent of the patient’s cancer or anti-cancer therapy status).

As previously discussed, several mechanisms might cause variations in eGFR; might they be true or spurious alterations? We believe that further studies assessing the value of the available kidney function biomarkers are warranted in cancer patients.

## Conclusions

Abemaciclib, a CDK4/6 inhibitor approved for the treatment of metastatic luminal breast cancer is very frequently associated with elevations in sCr, although this does not seem to represent true kidney injury. Cancer patients are often complex, possibly with several morbidities, and under treatment with drugs that can affect renal function. Healthcare providers should be aware of the known effect of abemaciclib on spurious sCr elevation but should not dismiss the possibility of true kidney injury associated with the drug. Furthermore, this case raises questions about the value of other biomarkers, such as sCysC, and their caveats in assessing GFR in cancer patients, under abemaciclib.

Alternative measures of assessment of GFR are recommended; in our case, direct measurement with a renogram was necessary and showed significant differences to the eGFR calculated with sCr and sCysC, having a notable impact on the maintenance of treatments that would have otherwise been limited by renal impairment. Additional research into the validity of sCysC testing in cancer patients receiving abemaciclib is necessary.
